# Harmonizing the COVID-19 sample biobanks: Barriers and opportunities for standards, best practices and networks

**DOI:** 10.1016/j.bsheal.2022.06.003

**Published:** 2022-08

**Authors:** Balwir Matharoo-Ball, Mbayame Diop, Zisis Kozlakidis

**Affiliations:** a Director of Biobank, G42 Healthcare, Abu Dhabi, United Arab Emirates; b Université Cote d’Azur, Nice 06100, France; c International Agency for Research on Cancer, World Health Organization, 69372 Lyon CEDEX 08, France

**Keywords:** Biobanks, COVID-19, Biobank standards, Biobank best practices, Biobank networks

## Abstract

The coronavirus disease 2019 (COVID-19) pandemic has highlighted the practice of infectious diseases biobanking, as well as existing challenges and opportunities. Thus, the future of infectious diseases biobanking in the post-pandemic era, shall not be an “entry-level version” of its counterpart in non-communicable diseases and large population cohorts, but incorporate the lessons learned. Biobanks constitute a critical research infrastructure supported by harmonized practices through the implementation of international standards, and perceived within the broader scope of healthcare's intersection with research. This perspective paper considers the barriers in biobanking and standardization of practices, as well as the emerging opportunities in the field.

## Highlights

• The COVID-19 pandemic has highlighted the practice of infectious diseases biobanking, as well as existing challenges and opportunities.
• Governance frameworks allowing for the sharing of materials and data, the plurarity of sample collection and storing SOPs and the adherence to biosafety level requirements were highlighted as potential operational barriers.
• The availability of an increased number of purpose-built biobanks with harmonized SOPs, allows for scalability of response in times of crisis.
Collaborations of infectious disease biobanks with existing vertical national and regional efforts provide synergistic opportunities that can positively impact biobank sustainability, staff training and retention.

## 1 Introduction

Infectious diseases, such as coronavirus disease 2019 (COVID-19), may become major global health threats with far-reaching consequences. The COVID-19 pandemic, within a short period of time, has caused heavy damage to both global public health security and human health [1]. Biological samples, such as blood and saliva samples, from COVID-19 positive patients are critical in understanding and researching on the disease if accompanied by relevant clinical data; and, because such associations between a pathogen and the development of disease are often weak, samples may be needed in large quantities. Thus, if more, well-characterized, high-quality samples are available through biobanks, research will advance faster and improve upon the delivery of healthcare. Therefore, biobanking becomes a key element to the success of future treatments, relied upon standardized tissue collection for improved scientific quality [2], [3].

In the same manner, the harmonization of practices between different biobanks will facilitate the sharing and comparative analyses of such samples and foster regional and international collaborations between researchers. This harmonization includes the data aspects, as associated data would also need to be shared and/or be amenable to comparisons. In addressing the need of biobank harmonization, international agencies (e.g., the Organisation for Economic Cooperation and Development [OECD], the International Agency for Research on Cancer [IARC] and International Organization for Standardization [ISO]) and professional infrastructures (e.g., the Biobanking and Biomolecular resources Research Infrastructure [BBMRI] and the International Society for Biological and Environmental Repositories [ISBER]) have developed guidelines, standards, and best practices for biobanking. These documents provide scientific, operational, ethical, and legal guidelines and requirements. They include the ISBER Best Practices for Biorepositories (ISBER BP) (latest, 4th edition in 2018) [4], the OECD Guidance for Human Genetic Research Databases (2006) [5], the IARC Common Minimum Technical Standards and Protocols for Biobanks Dedicated to Cancer Research (2017) (IARC CMTS) [6], and the ISO standard 20387:2018-Biotechnology-Biobanking-General Requirements for Biobanking (ISO20387) [7]. While these documents do not specifically focus on infectious disease, rather the general practice of biobanking, their implementation anticipates increasing the level of professionalism in biobanking [8].

Using COVID-19 as an example, this manuscript identifies the barriers and opportunities for biobanking harmonization and describes how to maximize and effectively use these limited and precious human biological resources; and how to build a scientific, systematic, and standardized biobank for infectious diseases. Thus, the aspects considered here, relate to scientific research for the prevention and control of infectious diseases in general, and the associated biological security aspects.

## 2 Barriers in biobanking and standardization of practices

A barrier is defined as any factor that constitutes an obstacle in the process of harmonizing practices in biobanking. Sharing is defined as the biobanking process for supplying samples with or without data, or only data to those requesting it [9]. Within biobanking, the discussion has focused on the relevant international norms, namely the 2005 International Health Regulations (IHR) [10], the 2011 Pandemic Influenza Preparedness (PIP) Framework [11], and the 2010 Nagoya Protocol on Access to Genetic Resources and the Fair and Equitable Sharing of Benefits [12]. Yet, there remain important gaps in understanding the full governance spectrum, including the conditions under which sharing samples and/or is likely to become problematic. For example, such challenges might include informal norms, for instance those between scientists or networks of research institutions, and specific agreements between organizations, e.g., material transfer agreements (MTAs) or research contracts. Efforts attempting to address these barriers are firstly the recently announced WHO BIOHub in Spiez, (Switzerland) that will enable WHO Member States to share biomaterials in a safe mechanism [13] and also the work group within the European, Middle Eastern and African Society for Biobanking and Biopreservation (ESBB).

Once the governance framework is established, then timely access to pathogen samples and related data is a critical precondition in the efforts to identify and understand pathogens, and subsequently to develop appropriate medical countermeasures (e.g., diagnostics, drugs, vaccines). In the case of infectious disease pathogens, a common option is to be able to access the genomic sequence data as early as possible, thereby diminishing the need for access to physical samples. This has certainly been the case for the COVID-19 pandemic, where the sequence was published as early as was technically possible [14], while the distribution of physical samples was curated through established networks and supply infrastructures [15]. However, the latter was successful in the case of pre-existing formal and informal collaborations. In some cases, the absence of trusted collaborations led to slow, inefficient, and potentially detrimental barriers to access pathogens, which may be difficult to overcome quickly in times of crisis [16].

One of the challenges in implementing guidelines and standards in infectious diseases biobanks is the multiplicity of already established standard operating processes (SOPs). SOPs are mentioned commonly in the published literature, with only legal issues being cited more frequently [9]. The differences in procedures between biobanks are described as a challenge, and in some cases, established biobank's practices may be suboptimal, and therefore a barrier, regardless of the differences with other banks. The variety of methods for the storage and processing of samples was mentioned as a barrier, as preparation methods in different laboratories and countries may not be uniform, resulting to lack of compatibility [17] and thus impacting the availability of samples for international projects. Having said that, the recent introduction of the new wave of biobanking guidelines and ISO standard should address some of these challenges, though none of the published biobank best practices and ISO standards contain any sections that are specific to infectious diseases.

Lastly, biobanking of potentially infectious samples from patients, animals or the environment need to be performed according to their respective biosafety level requirements. There are four biosafety levels that are implemented and defined by the Centers for Disease Control and Prevention (CDC) [18]. Each biosafety level has specific containment controls, including practices, safety equipment, and facility safeguards to protect laboratory staff, the public and the environment from exposure to infectious biohazards. These biosafety levels dictate the type of operations that are allowed in a lab setting and play a significant role in the design of the facility. As such, not all biobanking facilities can afford the operational or institutional arrangements for all types of infectious disease agents, but a careful consideration needs to take place.

## 3 Emerging opportunities

On the other hand, the creation of increasing numbers of infectious disease biobanks that have harmonized practices, offers a number of opportunities. In particular, the value of infectious diseases biobanks relies on the availability, at a necessary scale, of high-quality biospecimens and related data in order to respond to emerging biological questions, and as exemplified by the clinical urgency of the current COVID-19 pandemic, making those samples available at a rapid pace. The availability of high volumes of specimens is also expected to support high-throughput -omics technologies, but such methods remain to be validated by regulatory agencies [19] or to be harmonized as much as possible in order to be impactful.

Furthermore, discussions regarding the financial viability of many sample collections in the post-COVID-19 era, as well as the degree of availability of those collections to industrial partners are ongoing [20]. A major leap forward can be achieved if challenges relating to staff training and retention, funding and scale (that were temporarily addressed for the needs of the COVID-19 pandemic response) can be re-considered post-pandemic, as part of the building back better healthcare agenda [21]. However, this would necessitate the synergistic efforts of multiple stakeholders—as has been demonstrated during the mobilization of resources and efforts for vertical programmes relating to Human Immunodeficiency Virus (HIV), Tuberculosis (TB), and malaria, as well as regional translational infrastructures such as the European Research Infrastructure on Highly Pathogenic Agents (ERINHA), the European Virus Archive – Global (EVAg), the European COVID-19 Data Platform, and others. Such plans require a good understanding of the healthcare challenges, as well as the harmonization of biobanking practices, so that any contribution to downstream research can be inclusive of multiple facets (from diagnostic microbiology to business models). Thus, it can become tailored to a successful implementation and linked to already existing and successful initiatives [22].

## 4 Conclusion

The future of infectious diseases biobanking post-COVID-19, shall not be an “entry-level version” of its counterpart in non-communicable diseases and large population cohorts. Conversely, it will have to become a purpose-built, well-conceived, cost-effective and efficient research infrastructure, primarily supported by harmonized practices through the implementation of international standards, and perceived within the broader scope of healthcare's intersection with research (e.g., the European Health Data Space (EHDS) 1 - healthcare and EHDS 2 - secondary use). In this way, infectious disease biobanks will become ready to assume their place at the frontline of infectious diseases research, understanding and potentially surveillance.

## Conflict of interest statement

The authors declare that there are no conflicts of interest. Where authors are identified as personnel of the International Agency for Research on Cancer/WHO, the authors alone are responsible for the views expressed in this article and they do not necessarily represent the decisions, policy or views of the International Agency for Research on Cancer/WHO.

## Author contributions

**Balwir Matharoo-Ball:** Formal Analysis, Writing – Original Draft. **Mbayame Diop:** Writing – Review & Editing, Writing – Original Draft. **Zisis Kozlakidis:** Conceptualization, Methodology, Supervision, Writing – Review & Editing.
